# Yeast Cell-Based Transport Assay for the Functional Characterization of Human 4F2hc-LAT1 and ‐LAT2, and LAT1 and LAT2 Substrates and Inhibitors

**DOI:** 10.3389/fmolb.2021.676854

**Published:** 2021-05-28

**Authors:** Satish Kantipudi, Dimitrios Fotiadis

**Affiliations:** Institute of Biochemistry and Molecular Medicine, University of Bern, Bern, Switzerland

**Keywords:** amino acid transporter, inhibitor, LAT1, LAT2, JPH203, *Pichia pastoris*, SLC7, Transport Assay

## Abstract

In mammalian cells, the L-type amino acid transporters (LATs) LAT1 (SLC7A5) and LAT2 (SLC7A8) form heterodimeric amino acid transporters (HATs) with the ancillary protein 4F2hc and are involved in the cellular uptake of specific amino acids. The HAT 4F2hc-LAT1 is found upregulated in various cancer cell types, while 4F2hc-LAT2 is a transporter for non-cancer cells. Preclinical studies have highlighted that 4F2hc-LAT1 plays an important role in tumor progression representing a valid anticancer target. Consequently, current research is focusing on the development of potent and specific human 4F2hc-LAT1 inhibitors. On the other hand, 4F2hc-LAT2 is emerging as target of other diseases, thus also gaining clinical interest. To determine affinity and specificity of substrates and inhibitors for 4F2hc-LAT1 or 4F2hc-LAT2, robust transport cell assays are indispensable. We have optimized and validated a transport assay using cells of the methylotrophic yeast *Pichia pastoris* stably overexpressing the human HATs 4F2hc-LAT1 or -LAT2, and the LATs LAT1 or LAT2 alone. The radioligand [^3^H]L-leucine was used as reporter and the substrates L-leucine, triiodothyronine (T3) and thyroxine (T4) as well as the inhibitors BCH and JPH203 (KYT-0353) for assay validation. Obtained half-maximal inhibitory concentrations also provided new insights, e.g., into the LAT specificity of the potent inhibitor JPH203 and on the potency of the thyroid hormones T3 and T4 to inhibit transport through human 4F2hc-LAT2. The LAT1 and LAT2 assays are of particular interest to determine possible implications and influences of 4F2hc in ligand binding and transport. In summary, the presented assays are valuable for characterization of ligands, e.g., towards 4F2hc-LAT1 specificity, and can also be applied for compound screening. Finally, our established approach and assay would also be applicable to other HATs and LATs of interest.

## Introduction

Amino acids have diverse and essential roles in cell function, e.g., for protein synthesis, metabolism, signal transduction, neural transmission, and cellular growth and proliferation. Transport of amino acids across biological membranes is mediated by amino acid transporters, which are embedded in lipid bilayers of cells (Christensen, 1990; McGivan and Pastor-Anglada, 1994). Malfunction, absence or overexpression of amino acid transporters can affect homeostasis in the body leading to human diseases. The solute carrier (SLC) superfamily includes currently eleven families containing amino acid transporters (Kandasamy et al., 2018). The SLC7 family of amino acid transporters consists of fifteen genes and is split into two subgroups: the cationic amino acid transporters (CATs) and the L-type amino acid transporters (LATs) (Verrey et al., 2004; Fotiadis et al., 2013). CATs comprise the *SLC7A1-A4* and *SLC7A14* genes, and LATs the *SLC7A5-A11, Slc7a12, SLC7A13,* and *Slc7a15* genes (Fotiadis et al., 2013). In contrast to CATs, LATs are not glycosylated. For correct trafficking to the plasma membrane in mammalian cells, LATs associated with type II membrane N-glycoproteins from the SLC3 family, i.e., 4F2hc (SLC3A2; CD98) and rBAT (SLC3A1) (Palacin and Kanai, 2004). These ancillary proteins (the heavy chains) are covalently connected to the corresponding LATs (the light subunits) through a conserved disulfide bridge to form heterodimeric amino acid transporters (HATs) (Chillaron et al., 2001; Wagner et al., 2001; Palacin and Kanai, 2004; Verrey et al., 2004; Fotiadis et al., 2013). The light subunits are the catalytic subunits of HATs (Reig et al., 2002; Rosell et al., 2014; Napolitano et al., 2015).

LAT1 (SLC7A5) and LAT2 (SLC7A8) are isoforms of the system L of amino acid transporters requiring the heavy chain 4F2 (4F2hc) for functional expression at the plasma membrane (Kanai et al., 1998; Pineda et al., 1999; Segawa et al., 1999). Furthermore, we recently showed that 4F2hc can modulate the substrate affinity and specificity of the light chains LAT1 and LAT2 (Kantipudi et al., 2020). In addition to these two LAT specific functions, the ancillary protein 4F2hc has multifunctional roles such as in cell adhesion, cell fusion, integrin signaling and regulation of macrophage activation via galectin-3 (Fenczik et al., 1997; Tsurudome and Ito, 2000; Feral et al., 2005; MacKinnon et al., 2008). 4F2hc-LAT1 is expressed in different tissues and organs (e.g., brain, ovary, placenta and testis), and in relatively high levels at the blood-brain barrier and in several types of tumors (Fotiadis et al., 2013; Scalise et al., 2018; Häfliger and Charles, 2019). The location and high expression levels make 4F2hc-LAT1 an interesting vehicle for drug delivery into the brain and for cancer cell targeting (Häfliger and Charles, 2019; Puris et al., 2020). In cancer cells, 4F2hc-LAT1 provides neutral and essential amino acids for nutrition and regulation of the mTOR signaling pathway (Nicklin et al., 2009). Thus, inhibition of this HAT represents a valid approach to block migration and invasion of cancer cells, and to induce apoptosis. In contrast, 4F2hc-LAT2 is ubiquitously expressed in the human body and highly expressed in polarized epithelia suggesting a major role of this HAT in transepithelial transport of amino acids (Bröer, 2008; Fotiadis et al., 2013). Thus, both transporters have evolved towards specific functions, e.g., LAT1 for uptake of specific amino acids into growing cells, and LAT2 towards normal cell-type and transcellular amino acid transport.

LAT1 and LAT2 are sodium-independent transporters that exchange substrates across membranes with a one-to-one stoichiometry (Verrey et al., 2004; Fotiadis et al., 2013). The substrate specificities of both HATs are comparable, but 4F2hc-LAT2 accepts in addition to large neutral also small neutral amino acids (Pineda et al., 1999; Rossier et al., 1999; Meier et al., 2002). Other substrates of 4F2hc-LAT1 and -LAT2 represent amino acid derivatives such as the thyroid hormones T3 and T4 (Friesema et al., 2001; Zevenbergen et al., 2015). The compound 2-aminobicyclo-(2,2,1)-heptane-2-carboxylic acid (BCH) (Kim et al., 2008) was described as specific inhibitor of system L inhibiting both, 4F2hc-LAT1 and -LAT2 (Kanai et al., 1998; Segawa et al., 1999). On the other hand, the tyrosine-based JPH203 (KYT-0353) molecule was reported as a competitive, potent and highly specific 4F2hc-LAT1 inhibitor with strong inhibitory effects on the growth of different cancer cells (Oda et al., 2010; Yun et al., 2014; Häfliger et al., 2018). Therefore, transport inhibitors with high specificity towards 4F2hc-LAT1 but not -LAT2 represent promising drug candidates for cancer therapy and diagnosis. In crescentic glomerulonephritis pathogenesis, LAT2 was shown to be upregulated activating the mTORC1 pathway (Kurayama et al., 2011). Thus, LAT2-specific inhibitors might also be interesting and considered therapeutically for crescentic glomerulonephritis and other emerging LAT2-related diseases. Towards discovery of potent and selective inhibitors against 4F2hc-LAT1 or -LAT2, robust assays for ligand screening and functional characterization using cells overexpressing corresponding LATs separately are crucial. Establishment of mammalian cell lines for stable expression of 4F2hc-LAT1 and -LAT2 is not straight-forward since most host cell lines express HATs endogenously. As a consequence, the activity of the exogenous LAT is difficult to distinguish from the endogenous one and the limited endogenous pool of 4F2hc is used for both, endogenous and exogenous LATs, thus introducing ambiguities in the assays. Khunweeraphong *et al.* reported the establishment of stable HEK293 cell lines expressing exogenous LAT1 or LAT2, and using endogenous 4F2hc of the cells to form HATs (Khunweeraphong et al., 2012). HEK293 cells indicated reduced backgrounds of amino acid transport, e.g., reduced contamination of endogenous LAT1 activity (Khunweeraphong et al., 2012), and other advantages compared to the murine S2 cells previously used in a similar endeavor (Morimoto et al., 2008).

The methylotrophic yeast *Pichia pastoris* represents a well-established system for the expression of recombinant human membrane proteins (Byrne, 2015). Cholesterol and derivatives thereof were shown to play an important role in function and stability of LATs (Meury et al., 2014; Dickens et al., 2017; Cosco et al., 2020). Yeasts such as *P. pastoris* produce ergosterol (Nes et al., 1978) providing a valuable cholesterol derivate for interaction with heterologously expressed membrane proteins. Towards establishment of a robust assay for ligand screening and functional characterization, we have optimized, applied and validated a previously reported radioligand assay using *P. pastoris* overexpressing human 4F2hc-LAT1 or -LAT2, and the substrate [^3^H]L-leucine as radioligand. In contrast to the previously reported HEK293 cell assay (Khunweeraphong et al., 2012), 4F2hc is co-expressed with LAT1 or LAT2 in the *Pichia*-based assay, thus not limiting 4F2hc availability and boosting expression of HATs (Costa et al., 2013; Rosell et al., 2014; Kantipudi et al., 2020). Interestingly, and in absence of 4F2hc co-expression, *Pichia* is also able to express functionally the light subunits LAT1 and LAT2 alone (Costa et al., 2013; Rosell et al., 2014; Kantipudi et al., 2020). This allows evaluating possible contributions of the heavy chain 4F2hc on ligand binding and transport inhibition through selected substrates and inhibitors.

## Materials and Methods

### Cloning and Expression in *P. pastoris* of Human 4F2hc-LAT1, LAT1, 4F2hc-LAT2 and LAT2

Cloning of the human HATs and LATs into the pPICZB vector (Thermo Fisher Scientific, Waltham, MA, United States), electro-transformation of competent *P. pastoris* strain KM71H cells (Thermo Fisher Scientific, Waltham, MA, United States) and selection of clones with high protein expression levels was performed as described in detail previously: see for 4F2hc-LAT1 and LAT1 (Kantipudi et al., 2020), and for 4F2hc-LAT2 and LAT2 (Costa et al., 2013). Cell growth and expression conditions of *Pichia* clones overexpressing human 4F2hc-LAT1, LAT1, 4F2hc-LAT2 or LAT2, and untransformed *P. pastoris* KM71H cells (control) was conducted according to Kantipudi *et al.* (Kantipudi et al., 2020). Resulting cells were resuspended in transport buffer (150 mM choline chloride, 1 mM MgCl_2_, 1 mM CaCl_2_, 10 mM Tris-HEPES, pH 7.4) containing 50% (v/v) glycerol and the OD_600_ was adjusted to 40. Cells were stored at −18°C until further use.

### [^3^H]L-Leucine Radioligand Transport Assay

**FIGURE 1:**
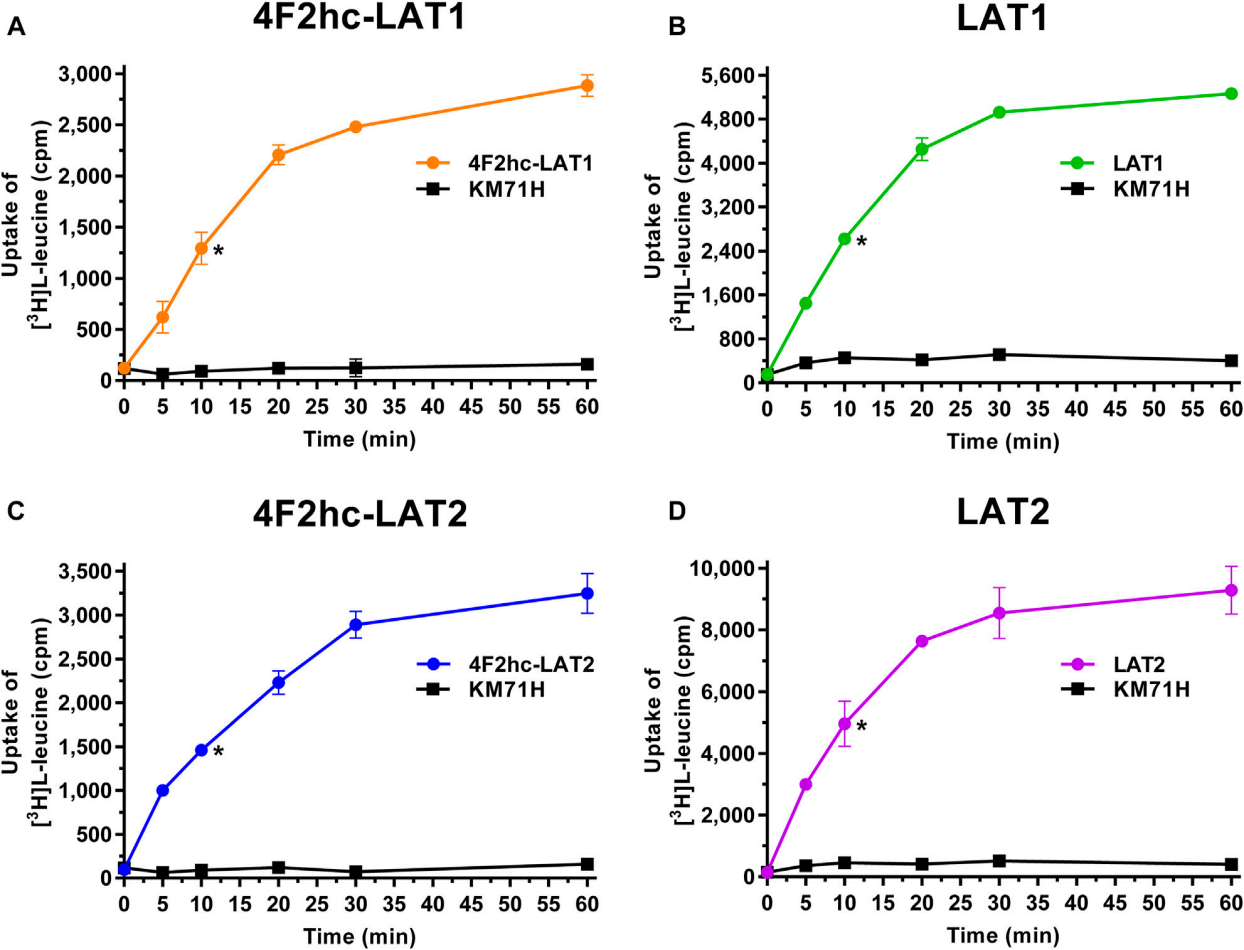
Time-dependent uptake of 0.1 µM [^3^H]L-leucine into *P. pastoris* KM71H cells expressing 4F2hc-LAT1 [**(A)**; orange], LAT1 [**(B)**; green], 4F2hc-LAT2 [**(C)**; blue], or LAT2 [**(D)**; violet]. [^3^H]L-leucine uptake into untransformed *P. pastoris* KM71H cells is shown in black. Asterisks indicate the 10 min time points located in the linear regimes chosen for the subsequently presented transport inhibition experiments (Figures 2–6). Data points are represented as mean ± standard deviation from a representative experiment in triplicate. If not visible, error bars are smaller than symbols.

**FIGURE 2:**
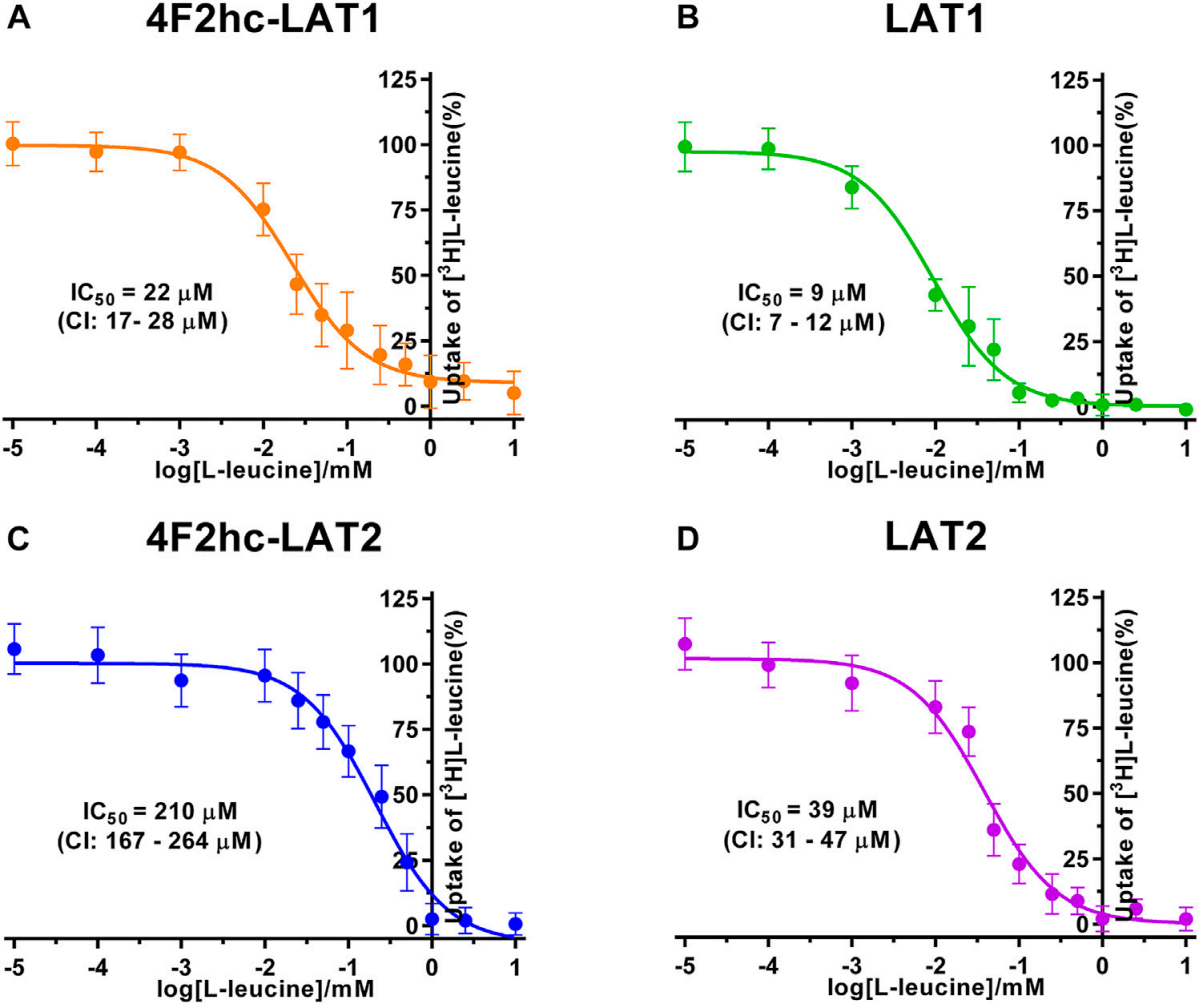
IC_50_ determination of L-leucine for human 4F2hc-LAT1 [**(A)**; orange], LAT1 [**(B)**; green], 4F2hc-LAT2 [**(C)**; blue] and LAT2 [**(D)**; violet] using *P. pastoris* KM71H cells expressing the corresponding transporter variants. Determined IC_50_ values and 95% confidence intervals (CIs) are indicated. Mean ± standard deviation of normalized data from three independent experiments, each at least in triplicate, are shown. If not visible, error bars are smaller than symbols.

**FIGURE 3:**
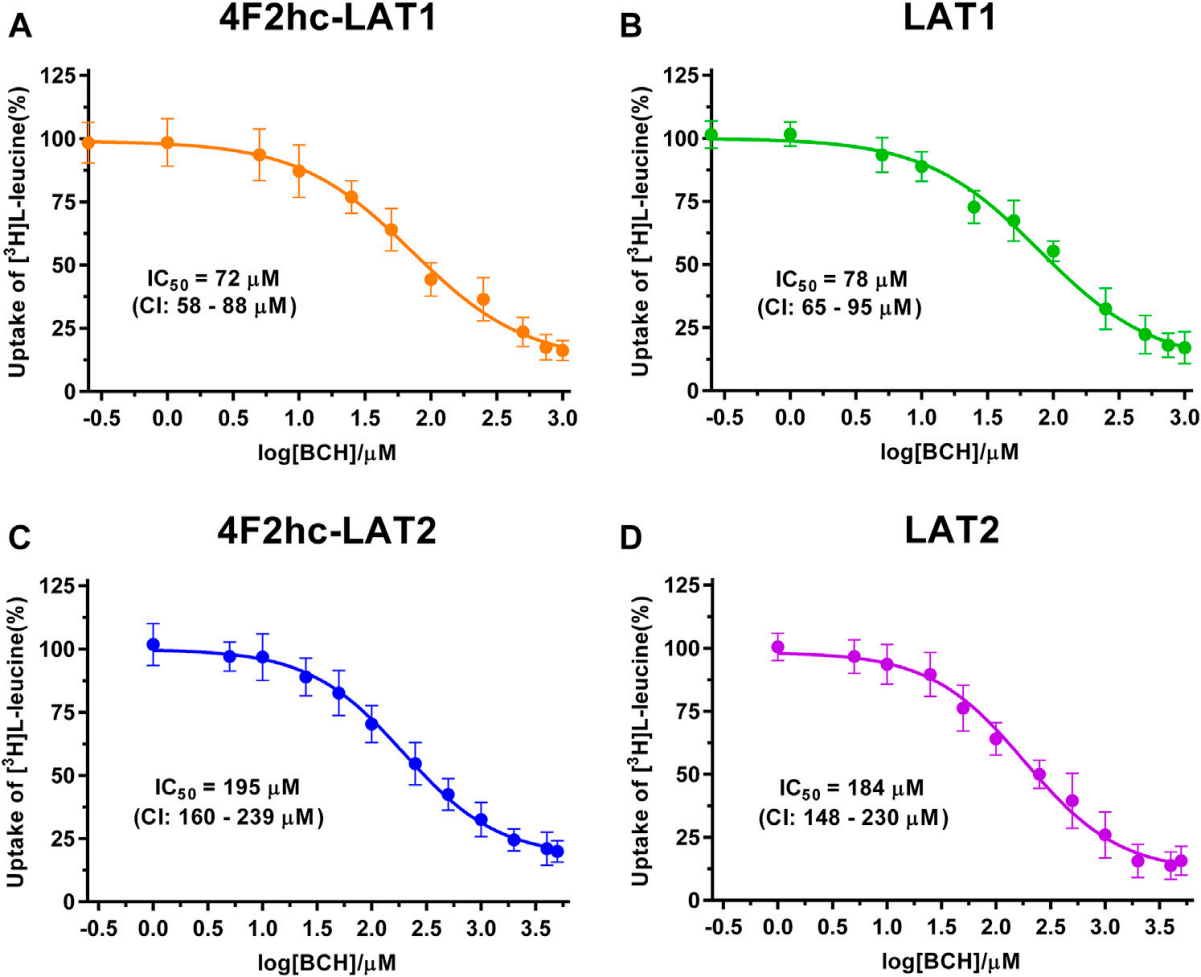
IC_50_ determination of BCH for human 4F2hc-LAT1 [**(A)**; orange], LAT1 [**(B)**; green], 4F2hc-LAT2 [**(C)**; blue] and LAT2 [**(D)**; violet] using *P. pastoris* KM71H cells expressing the corresponding transporter variants. Determined IC_50_ values and 95% confidence intervals (CIs) are indicated. Mean ± standard deviation of normalized data from three independent experiments, each at least in triplicate, are shown. If not visible, error bars are smaller than symbols.

For transport studies, 1 ml of thawed *P. pastoris* cells (OD_600_ 40) expressing the corresponding transporter were diluted in 50 ml transport buffer and pelleted by centrifugation (3,000 × *g*, 15 min, room temperature). The pellet was then washed by resuspending it in 50 mL transport buffer and by repeating the washing procedure (centrifugation and resuspension) two times. Finally, the cell pellet was resuspended in 2 ml of transport buffer and incubated for 20 min at 30°C under agitation (300 rpm, Multitron, Infors HT, Bottmingen, Switzerland). The yeast suspension density was adjusted with transport buffer to an OD_600_ of 1.875 (4F2hc-LAT1, LAT1, 4F2hc-LAT2 or LAT2, and untransformed *P. pastoris* KM71H cells). All transport experiments were performed in a reaction volume of 100 µL. For time-dependent [^3^H]L-leucine uptake experiments (Figure 1), the reaction mixture contained 40 µL cell suspension and 60 µL substrate master mix [0.167 µM L-leucine spiked with [^3^H]L-leucine (American Radiolabeled Chemicals, St. Louis, MO, United States)] with a specific activity of 20 Ci/mmol resulting in a final L-leucine concentration of 0.1 µM. IC_50_ determinations of selected compounds for the transporters 4F2hc-LAT1, LAT1, 4F2hc-LAT2, or LAT2 were performed using 40 µL cell suspension, 50 µL of competitor solution at different concentrations and 10 µL substrate master mix [1 µM L-leucine spiked with [^3^H]L-leucine (American Radiolabeled Chemicals, St. Louis, MO, United States)] with a specific activity of 20 Ci/mmol resulting in a final L-leucine concentration of 0.1 µM. For L-leucine and BCH (2-aminobicyclo-(2,2,1)-heptane-2-carboxylic acid) IC_50_ experiments, the competitor solutions were at concentrations of 0.01−10,000 µM L-leucine (4F2hc-LAT1, LAT1, 4F2hc-LAT2, and LAT2), and 0.25−1,000 µM BCH (4F2hc-LAT1 and LAT1) and 1−5,000 µM BCH (4F2hc-LAT2 and LAT2)–see corresponding graphs in Figures 2, 3 for specific concentrations used. For determining the IC_50_ values of hydrophobic compounds such as JPH203 [(S)-2-amino-3-(4-((5-amino-2-phenylbenzo [d] oxazol-7-yl)-methoxy)-3,5-dichlorophenyl)-propanoic acid] also known as KYT-0353 (MedChemExpress, Monmouth Junction, NJ, United States), and the thyroid hormones T3 (triiodothyronine) and T4 (thyroxine) (Sigma, St. Louis, MO, United States), 40 µL of cell suspension was incubated with 50 µL of competitor solutions at different concentrations for 60 min at 25°C under agitation (500 rpm, Thermomixer compact, Eppendorf, Hamburg, Germany). The uptake was initiated by adding 10 µL substrate master mix [1 µM L-leucine spiked with [^3^H]L-leucine (American Radiolabeled Chemicals, St. Louis, MO, United States)] with a specific activity of 20 Ci/mmol to the preincubated 90 µL of reaction volume resulting in a final L-leucine concentration of 0.1 µM. Final ligand concentrations in 100 µL were 0.001−250 µM (JPH203, T3, and T4). The competitors were prepared in 100% (v/v) DMSO. The final DMSO concentration was always 0.5% (v/v), independently of which final substrate or inhibitor concentration was used in the assay. Control samples contained the same concentration of DMSO. Final OD_600_ values in uptake experiments were 0.75 for all cells (4F2hc-LAT1, LAT1, 4F2hc-LAT2 or LAT2 expressing cells) and untransformed *P. pastoris* KM71H cells. All transport reactions were done in 2 ml reaction tubes (Eppendorf, Hamburg, Germany) at 25°C under agitation (1,000 rpm, Thermomixer compact, Eppendorf, Hamburg, Germany). Uptakes were terminated after 10 min (uptake time) for all cells by adding 600 µL of pre-chilled transport buffer. Cells were rapidly separated from the buffer by transferring the stopped reactions on a 96-well 0.66 mm glass fiber filter plate (Corning FiltrEX, Corning, NY, United States) and vacuum filtration. Each well was washed with 2 ml of ice-cold transport buffer to remove free radioligand. The plate was then dried overnight at 37°C, and the backside was sealed with a BackSeal (PerkinElmer, Waltham, MA, United States). The trapped radioligand was released by addition of 200 µL scintillation cocktail (MicroScint 40, PerkinElmer, Waltham, MA, United States) to each well, and the plate topside was sealed with Topseal^TM^-A Plus (PerkinElmer, Waltham, MA, United States), followed by incubation for 30 min at 25°C and 1,000 rpm (Thermomixer compact, Eppendorf, Hamburg, Germany). Counts were measured in each well for 2 min with a scintillation counter (TopCount NXT, PerkinElmer, Waltham, MA, United States).

### Data Analysis, Curve Fitting and Statistics

Experiments were performed at least in triplicate. For data analysis, the signal of the untransformed *P. pastoris* cells was subtracted from the transporter signal to obtain the net transport signal. Data from three independent experiments were taken. In each of these experiments, the net transport signals were averaged and the half maximal inhibitory concentration (IC_50_) values of homologous (L-leucine) and heterologous (BCH, JPH203, T3, and T4) L-leucine transport competition experiments were determined by fitting a sigmoidal model curve to these data. Every experimental data point was then individually normalized using the corresponding upper plateau values (i.e., the fitted upper plateau value that corresponds to 100%). Single, normalized data points from the three independent experiments were averaged and a sigmoidal model curve was fitted to the data in order to obtain the IC_50_ values. Prism6 (GraphPad Software) was used for data analysis.

## Results

Human 4F2hc-LAT1, LAT1, 4F2hc-LAT2 or LAT2 were expressed in the methylotrophic yeast *P. pastoris*. We showed in previous reports that not only the HATs 4F2hc-LAT1 and -LAT2, but also the light subunits LAT1 and LAT2 in absence of ancillary protein are properly folded, correctly trafficked to the plasma membrane and functional in *P. pastoris* (Rosell et al., 2014; Kantipudi et al., 2020).

**FIGURE 4:**
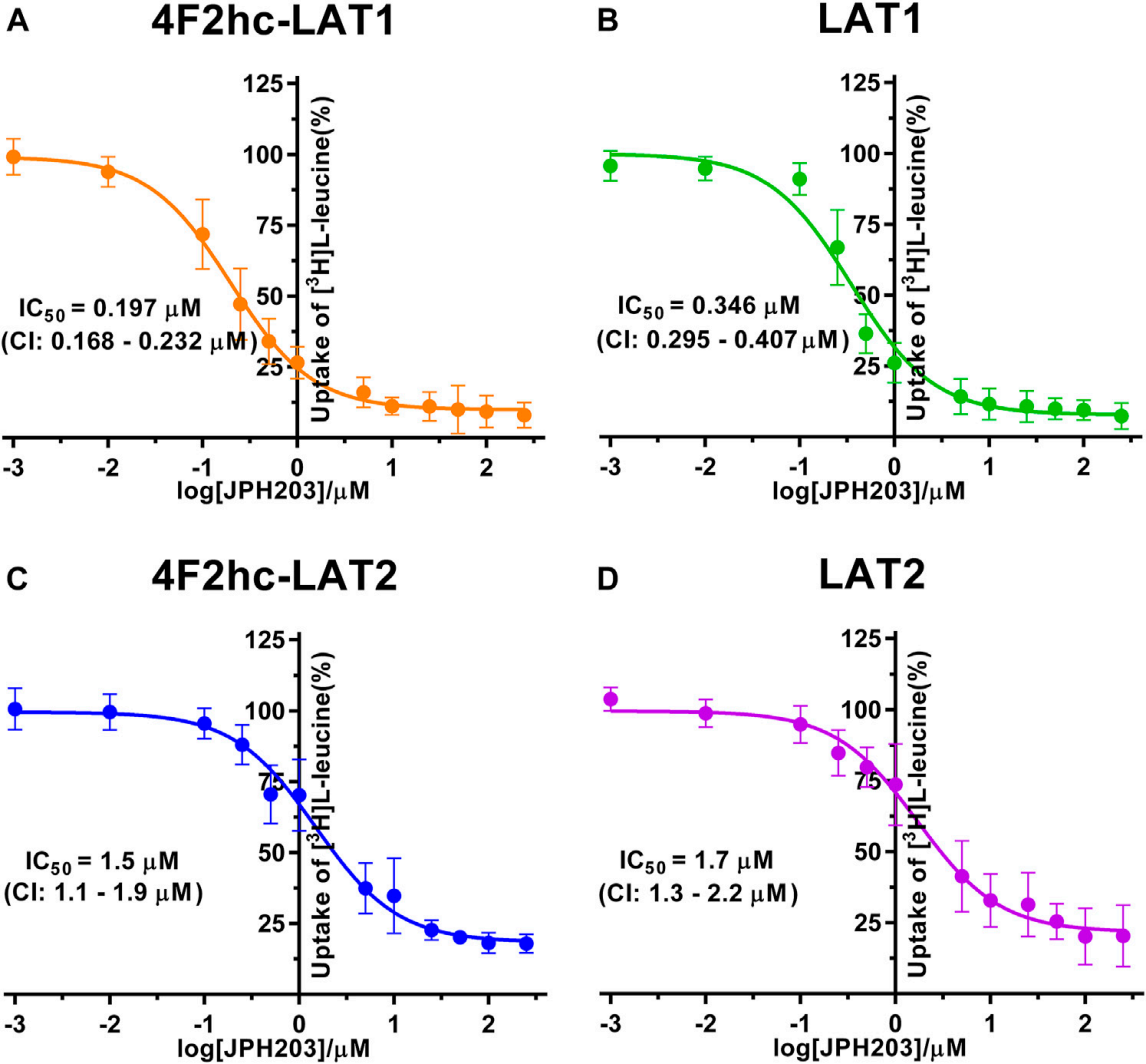
IC_50_ determination of JPH203 for human 4F2hc-LAT1 [**(A)**; orange], LAT1 [**(B)**; green], 4F2hc-LAT2 [**(C)**; blue] and LAT2 [**(D)**; violet] using *P. pastoris* KM71H cells expressing the corresponding transporter variants. Determined IC_50_ values and 95% confidence intervals (CIs) are indicated. Mean ± standard deviation of normalized data from three independent experiments, each at least in triplicate, are shown. If not visible, error bars are smaller than symbols.

**FIGURE 5:**
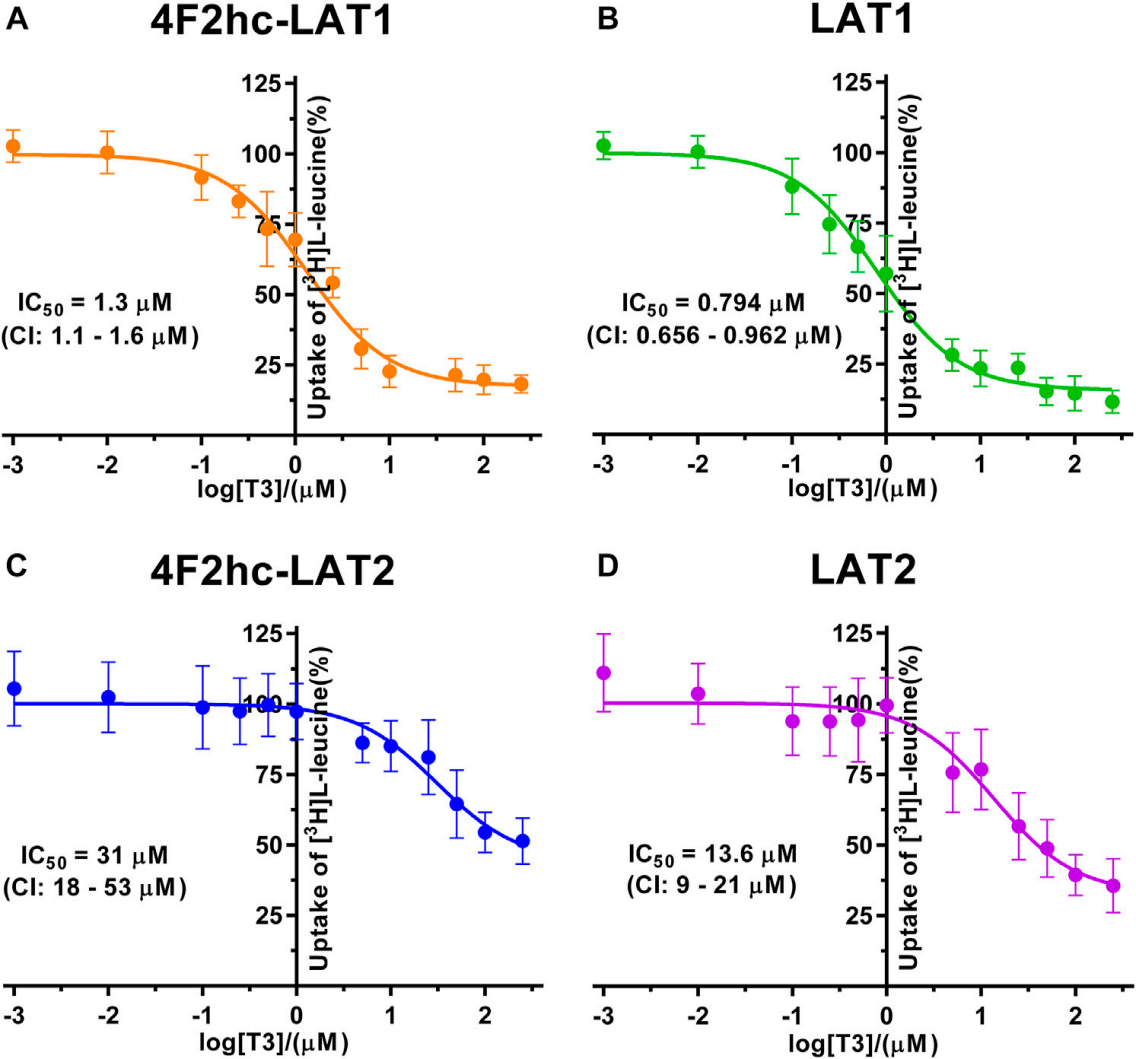
IC_50_ determination of T3 for human 4F2hc-LAT1 [**(A)**; orange], LAT1 [**(B)**; green], 4F2hc-LAT2 [**(C)**; blue] and LAT2 [**(D)**; violet] using *P. pastoris* KM71H cells expressing the corresponding transporter variants. Determined IC_50_ values and 95% confidence intervals (CIs) are indicated. Mean ± standard deviation of normalized data from three independent experiments, each at least in triplicate, are shown. If not visible, error bars are smaller than symbols.

**FIGURE 6:**
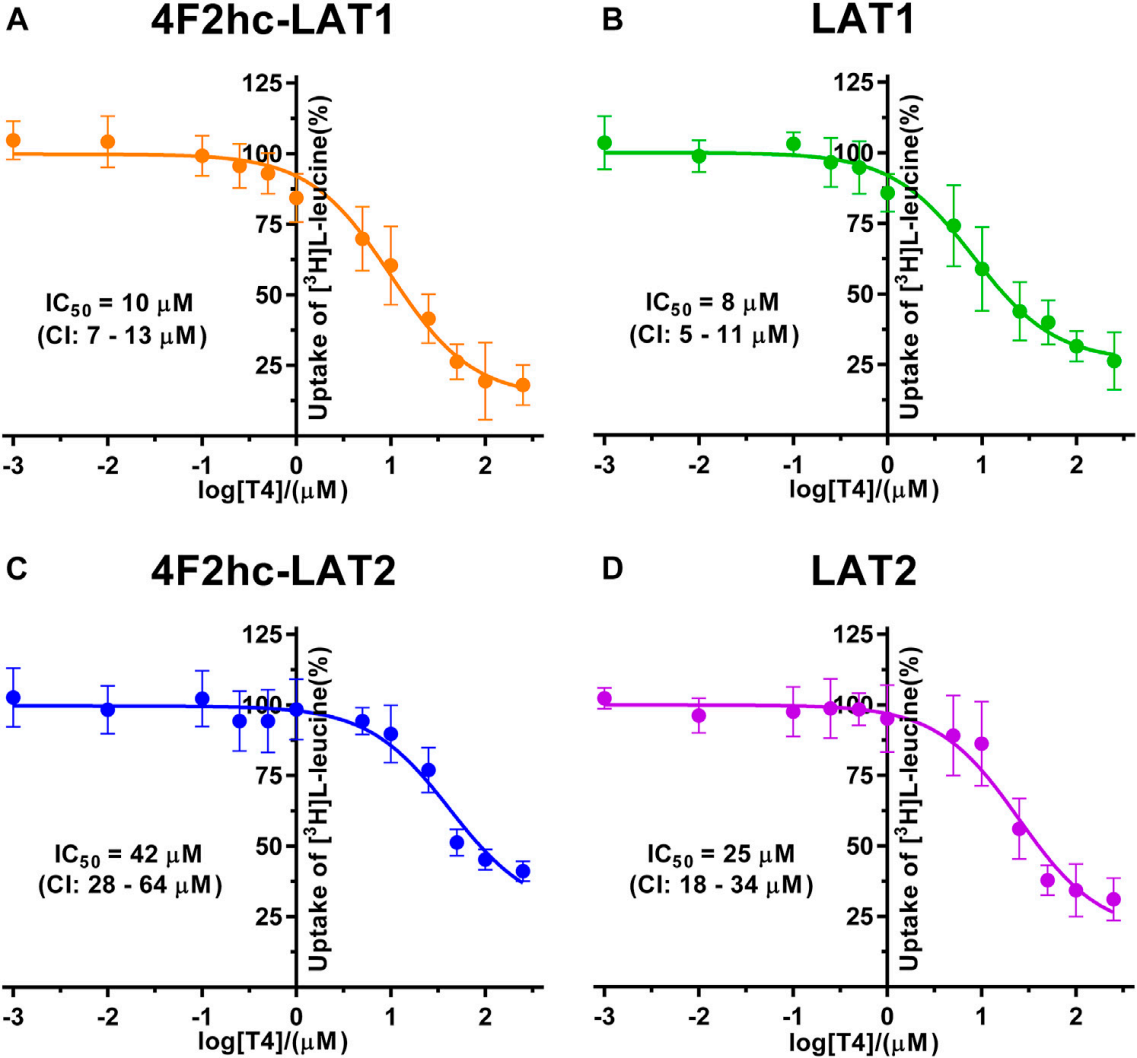
IC_50_ determination of T4 for human 4F2hc-LAT1 [**(A)**; orange], LAT1 [**(B)**; green], 4F2hc-LAT2 [**(C)**; blue] and LAT2 [**(D)**; violet] using *P. pastoris* KM71H cells expressing the corresponding transporter variants. Determined IC_50_ values and 95% confidence intervals (CIs) are indicated. Mean ± standard deviation of normalized data from three independent experiments, each at least in triplicate, are shown. If not visible, error bars are smaller than symbols.

Transport activities were determined by measuring the uptake of the substrate [^3^H]L-leucine into *Pichia* cells at OD_600_ 0.75 expressing the corresponding HAT or LAT. Time-course experiments showed clear HAT- and LAT-dependent transport activities, which were much higher than the [^3^H]L-leucine uptake into untransformed host cells (Figure 1). In all cases, saturation of the transport process was observed. Differences in radioligand transport (Figure 1) are due to different expression levels of corresponding recombinant LATs as estimated from V_max_/OD values using previously determined kinetic parameters (Kantipudi et al., 2020). Uptake assay times of 10 min (i.e., in the linear regimes–time points indicated by asterisks in Figure 1) and corresponding *Pichia* cells at OD_600_ 0.75 were taken for the subsequently presented experiments with 4F2hc-LAT1, LAT1, 4F2hc-LAT2, and LAT2 (Figures 2–6).

We determined the half-maximal inhibitory concentrations (IC_50_s) of L-leucine by homologous competition for all four transporter variants. Obtained IC_50_ values were 22 µM (4F2hc-LAT1), 9 µM (LAT1), 210 µM (4F2hc-LAT2), and 39 µM (LAT2) (Figure 2).

As previously reported (Kantipudi et al., 2020), comparison of IC_50_s indicated a modulatory effect of the heavy subunit 4F2 on light subunits, which is most striking between 4F2hc-LAT2 and LAT2 (Figure 2). After validation of the yeast-cell based transport assay with the substrate L-leucine (Figures 1, 2), we pursued validation using the described system L transport inhibitor BCH (Kim et al., 2008) and the 4F2hc-LAT1 specific inhibitor JPH203 (Oda et al., 2010). We obtained IC_50_ values of 72 µM (4F2hc-LAT1), 78 µM (LAT1), 195 µM (4F2hc-LAT2), and 184 µM (LAT2) for BCH (Figure 3). These results indicated that the compound BCH inhibits both human HATs being about 2.7-fold more specific for 4F2hc-LAT1. Interestingly, and in contrast to L-leucine (Figure 2), LAT1 and LAT2 in absence of 4F2hc had comparable IC_50_s as their heterodimeric forms (Figure 3) indicating no significant influence of the ancillary protein on BCH binding to LATs of HATs.

For the second inhibitor, i.e., JPH203, IC_50_ values of 0.197 µM (4F2hc-LAT1), 0.346 µM (LAT1), 1.5 µM (4F2hc-LAT2) and 1.7 µM (LAT2) were obtained using our yeast cell-based assay (Figure 4). Clearly, the compound JPH203 is with its three-digit nanomolar IC_50_ value potent and about 7.6-fold more specific for 4F2hc-LAT1 compared with 4F2hc-LAT2. As observed also with BCH, IC_50_ values of JPH203 were comparable for 4F2hc-LAT1 and LAT1, and 4F2hc-LAT2 and LAT2, indicating no significant effect of the ancillary protein on inhibitor binding.

The thyroid hormones triiodothyronine (T3) and thyroxine (T4) represent substrates of 4F2hc-LAT1 and -LAT2 (Friesema et al., 2001; Zevenbergen et al., 2015). We selected these amino acid derivatives as substrates for further validation of our *P. pastoris* cell-based transport assay. For triiodothyronine, we obtained IC_50_ values of 1.3 µM (4F2hc-LAT1), 0.794 µM (LAT1), 31 µM (4F2hc-LAT2), and 13.6 µM (LAT2) (Figure 5). The inhibitory effect of T3 was considerably more pronounced for 4F2hc-LAT1 and LAT1 compared with 4F2hc-LAT2 and LAT2, e.g., about 24-fold lower IC_50_ value for 4F2hc-LAT1 in comparison with 4F2hc-LAT2.

Finally, we determined the IC_50_s of the thyroid hormone thyroxine using our yeast cell-based transport assay and obtained IC_50_ values of 10 µM (4F2hc-LAT1), 8 µM (LAT1), 42 µM (4F2hc-LAT2), and 25 µM (LAT2) (Figure 6). Similar to triiodothyronine, the thyroxine hormone also competed with [^3^H]L-leucine uptake through 4F2hc-LAT1, LAT1, 4F2hc-LAT2, and LAT2. The transport inhibitory effect and thus specificity of T4 was about four-times higher for 4F2hc-LAT1 compared with 4F2hc-LAT2. A comparable trend was observed for the light subunits LAT1 and LAT2. The presence of the heavy chain 4F2 had no marked or only a weak effect on the IC_50_ values of 4F2hc-LAT1 (10 µM) and 4F2hc-LAT2 (42 µM) compared to the light subunits LAT1 (8 µM) and LAT1 (25 µM) alone.

## Discussion

A cell-based transport assay using the methylotrophic yeast *P. pastoris* overexpressing the human HATs 4F2hc-LAT1 or -LAT2, and LATs LAT1 or LAT2, and the radiolabeled substrate [^3^H]L-leucine was optimized and validated using selected substrates and inhibitors. In contrast to our previous study (Kantipudi et al., 2020), we adjusted uptake time and OD_600_ for the four stably expressing *Pichia* clones to same conditions, i.e., to 10 min and OD_600_ 0.75 (Figure 1). For comparison and validation of the adjusted uptake conditions, IC_50_s were determined for the substrate L-leucine (Figure 2)–the here studied transporters have high affinities for L-leucine relative to the other proteinogenic amino acids (Kantipudi et al., 2020). The obtained IC_50_s for L-leucine (Figure 2) were comparable to previously published values using oocytes (Kanai et al., 1998; Friesema et al., 2001; Yanagida et al., 2001), and *P. pastoris* cells at OD_600_ 10 (4F2hc-LAT1, 4F2hc-LAT2, and LAT1) and OD_600_ 3 (LAT2), and uptake times of 10 min (4F2hc-LAT1, 4F2hc-LAT2, and LAT1) and 2 min (LAT2) (Kantipudi et al., 2020). The modulatory effect of the heavy chain 4F2 on the L-leucine affinity of light subunits, this being most pronounced for 4F2hc-LAT2 and LAT2 (Figures 2C,D), was also observed in line with our previous report (Kantipudi et al., 2020). Newly, we tested with the *P. pastoris* cell-based assay, described transport inhibitors, i.e., the system L and 4F2hc-LAT1 uptake inhibitors BCH (Kim et al., 2008) and JPH203 (Oda et al., 2010), respectively. The IC_50_s of BCH for the two HATs were comparable with values from the literature (Table 1) and almost identical to the IC_50_s from corresponding LATs in absence of 4F2hc (Figure 3). The latter indicated no contribution of the ancillary protein on BCH binding to light subunits. JPH203 was described as a competitive, potent and highly specific 4F2hc-LAT1 inhibitor (Endou et al., 2008; Oda et al., 2010). The authors of these two studies determined IC_50_s of 0.14 µM (Oda et al., 2010), and 0.19 and 0.2 µM (Endou et al., 2008) for human LAT1 HATs expressed in S2 cells derived from mouse renal proximal tubules (Morimoto et al., 2008) (Table 1). The IC_50_ of 0.197 µM for 4F2hc-LAT1 obtained with the here presented assay (Figure 4A) is similar to the values (0.14, 0.19, and 0.2 µM) determined for the same HAT using S2 cells (Table 1). Furthermore, most kinetic parameters for JPH203 and 4F2hc-LAT1 determined using other cell types than S2 cells were also in good agreement with the IC_50_ value from *P. pastoris* cells (Table 1). However, a marked difference is found between the IC_50_ of JPH203 determined for human 4F2hc-LAT2 using the *P. pastoris* (1.5 µM; Figure 4C) and the S2 cell-based assays (>10 µM (Oda et al., 2010) and almost no inhibition at 10 µM (Endou et al., 2008)). Furthermore, Oda *et al*. indicated an IC_50_(S2-LAT2)/IC_50_(S2-LAT1) ratio of >500, which would be indicative of a high specificity of JPH203 towards human 4F2hc-LAT1 and not 4F2hc-LAT2 (Oda et al., 2010). In strong contrast, the IC_50_(*Pichia*-4F2hc-LAT2)/IC_50_(*Pichia*-4F2hc-LAT1) ratio was only about 7.6, indicating a significantly more moderate specificity of JPH203 towards 4F2hc-LAT1. This finding was further corroborated independently using Pichia cells expressing the light subunits alone, which yielded a comparable IC_50_(*Pichia*-LAT2)/IC_50_(*Pichia*-LAT1) ratio of about 4.9. Future studies on the specificity of JPH203 towards 4F2hc-LAT1 and -LAT2 will clarify this ambiguity. For further validation of our yeast cell-based transport assay, we tested amino acid derivatives, specifically the thyroid hormones T3 and T4, which are substrates of 4F2hc-LAT1 and -LAT2 (Friesema et al., 2001; Zevenbergen et al., 2015). For 4F2hc-LAT1, T3 reflected a higher potency in transport inhibition than T4 (Figures 5A, 6A) in line with previous studies (Table 1). No kinetic parameters were found in the literature for T3 and T4 with 4F2hc-LAT2 for comparison. Thus, our IC_50_ for T3 and T4 represent first 4F2hc-LAT2 and LAT2 specific kinetic values (Figures 5C,D, 6C,D). Based on the obtained IC_50_s, the ancillary protein 4F2hc did not have an important influence on the binding of the two hormones to the light subunits (Figures 5, 6).

**TABLE 1:** Comparison of obtained with published 4F2hc-LAT1 and -LAT2 kinetic parameters.

Compound	4F2hc-LAT1 IC_50_, *K*_M_ or *K*_i_ (µM) of L-leucine uptake			References
	This work (IC_50_)	Kinetic parameters	Host	
L-leucine	22	38 (IC_50_)	*Pichia pastoris*	Kantipudi et al. (2020)
		25 ± 3 (*K*_M_)		
		46 (*K*_M_)	Oocytes	Friesema et al. (2001)
		18.1 ± 3.4 (*K*_M_)		Kanai et al. (1998)
		19.7 ± 4.1 (*K*_M_)		Yanagida et al. (2001)
BCH	72	72.17 ± 0.92 (IC_50_)	Saos2	Choi et al. (2017)
		78.8 ± 3.5 (IC_50_)		Kim et al. (2008)
		75.3 ± 6.7 (IC_50_)	KB cells	Kim et al. (2008)
		73.1 ± 4.5 (IC_50_)	C6 cells
		92.6 ± 8.9 (IC_50_)	YD-38	Yun et al. (2014)
		78.3 ± 12.1 (IC_50_)	HEK293	Khunweeraphong et al. (2012)
		132 ± 27.8 (IC_50_)	S2 cells	Morimoto et al. (2008)
JPH203	0.197	1.31 ± 0.27 (IC_50_)	Saos2	Choi et al. (2017)
		0.14 (IC_50_)	S2 cells	Oda et al. (2010)
		0.19 and 0.20 (IC_50_)		Endou et al. (2008)
		0.79 ± 0.06 (IC_50_)	YD-38	Yun et al. (2014)
		0.134 (IC_50_)	HT-29	Häfliger et al. (2018)
		0.177 (IC_50_)	SW1736	
		0.120 (IC_50_)	8505c	
		0.20 ± 0.03 (IC_50_)	KKU-055	Yothaisong et al. (2017)
		0.12 ± 0.02 (IC_50_)	KKU-213	
		0.25 ± 0.04 (IC_50_)	KKU-100	
T3	1.3	0.8 (*K*_M_)	Oocytes	Friesema et al. (2001)
		1.8 (*K*_M_)		Ritchie et al. (1999)
		1.7 ± 0.1 (*K*_i_)	S2 cells	Morimoto et al. (2008)
T4	10	7.9 (*K*_M_)	Oocytes	Friesema et al. (2001)
		6.3 (*K*_M_)		Ritchie et al. (1999)
		115 ± 2.0 (*K*_i_)	S2 cells	Morimoto et al. (2008)

Compound	4F2hc-LAT2 IC_50_, *K*_M_ or *K*_i_ (µM) of L-leucine uptake			References
	This work (IC_50_)	Kinetic parameters	Host	
L-leucine	210	221 (IC_50_)	*Pichia pastoris*	Kantipudi et al. (2020)
		249 ± 22 (*K*_M_)		
		220 (*K*_M_)	Oocytes	Pineda et al. (1999)
BCH	195	315 ± 68.6 (IC_50_)	S2 cells	Morimoto et al. (2008)
JPH203	1.5	>10 (IC_50_)	S2 cells	Oda et al. (2010)

## Conclusion

We have provided a robust yeast cell-based transport assay for the functional characterization of human 4F2hc-LAT1 and -LAT2, and LAT1 and LAT2 substrates and inhibitors. *P. pastoris* cells expressing HATs are valuable for determining kinetic parameters of ligands, e.g., potency and selectivity of transport inhibitors towards 4F2hc-LAT1 and -LAT2. Such kinetic information will help evaluating the structure-activity relationship of new ligands. Moreover, assays performed with yeast cells expressing light subunits provide kinetic parameters that, when compared with those of corresponding HATs, are of interest towards identification of possible effects of the ancillary protein 4F2hc on ligand affinity and specificity. First kinetic parameters on T3 and T4 were successfully provided for human 4F2hc-LAT2 and LAT2 using the here presented yeast cell-based transport assay. Finally, the established approach and assay could also be used for compound screening and applied to other HATs and LATs of interest.

## Data Availability

The raw data supporting the conclusions of this article will be made available by the authors, without undue reservation.
